# The Proliferative Role of Immune Checkpoints in Tumors: Double Regulation

**DOI:** 10.3390/cancers14215374

**Published:** 2022-10-31

**Authors:** Xi-Yang Tang, Zhong-Lin Luo, Yan-Lu Xiong, Jie Yang, An-Ping Shi, Kai-Fu Zheng, Yu-Jian Liu, Chen Shu, Nan Ma, Qiang Lu, Jin-Bo Zhao

**Affiliations:** 1 Department of Thoracic Surgery, Tangdu Hospital, Air Force Medical University, 569 Xinsi Road, Xi’an 710038, China; 2 Department of Cardiothoracic Surgery, Peace Hospital, Changzhi Medical College, 161 Jiefang East Street, Changzhi 046000, China; 3 Department of Radiology, Functional and Molecular Imaging Key Lab of Shaanxi Province, Tangdu Hospital, Fourth Military Medical University (Air Force Medical University), 569 Xinsi Road, Xi’an 710038, China; 4 Department of Ophthalmology, Tangdu Hospital, Air Force Medical University, Xi’an 710038, China

**Keywords:** immune checkpoint, immune response, proliferation, tumor, signaling pathway

## Abstract

**Simple Summary:**

Cancer remains a serious health problem owing to its high morbidity and mortality. Immunotherapy, represented by anti-programmed death-1D1/anti-PD-Ligand 1 treatments, has become one of the most important methods for cancer treatment. However, there are still some challenges in this method such as low response rate, limited therapeutic targets, and unclear underlying molecular mechanisms of immune checkpoints. Thus, in this review, we aim to focus on the proliferative role of fifteen immune checkpoints that occur in various tumors. This has provided more clarity on the functions and mechanisms of immune checkpoints in tumors, especially on their proliferation role. This may provide better insights in developing more therapeutic targets and strategies in tumor immunotherapy.

**Abstract:**

Cancer remains a serious social health problem, and immunotherapy has become the major treatments in tumor treatment. Additionally, improving the efficiency and safety of treatment is necessary. Further, more therapy targets are warranted for future tumor treatments. In this review, in addition to examining the currently recognized role of immune regulation, we focus on the proliferative role of 15 immune checkpoints in various tumors, including PD1, PD-L1, FGL1, CD155, CD47, SIRPα, CD276, IDO1, SIGLEC-15, TIM3, Galectin-9, CD70, CD27, 4-1BBL, and HVEM. We managed to conclude that various immune checkpoints such as PD1/PD-L1, FGL1, CD155, CD47/SIRPα, CD276, and SIGLEC-15 all regulate the cell cycle, and specifically through Cyclin D1 regulation. Furthermore, a variety of signal pathways engage in proliferation regulation, such as P13K, AKT, mTOR, and NK-κB, which are also the most common pathways involved in the regulation of immune checkpoint proliferation. Currently, only PD1/PD-L1, CD47/SIRPα, TIM3/Galectin-9, and CD70/CD27 checkpoints have been shown to interact with each other to regulate tumor proliferation in pairs. However, for other immune checkpoints, the role of their receptors or ligands in tumor proliferation regulation is still unknown, and we consider the enormous potential in this area. An increasing number of studies have validated the various role of immune checkpoints in tumors, and based on this literature review, we found that most of the immune checkpoints play a dual regulatory role in immunity and proliferation. Therefore, the related pathways in proliferation regulation can served the role of therapy targets in tumor therapy. Further, great potential is displayed by IDO1, SIGLEC-15, 4-1BBL, and HVEM in tumor proliferation regulation, which may become novel therapy targets in tumor treatment.

## 1. Introduction

**Figure 1:**
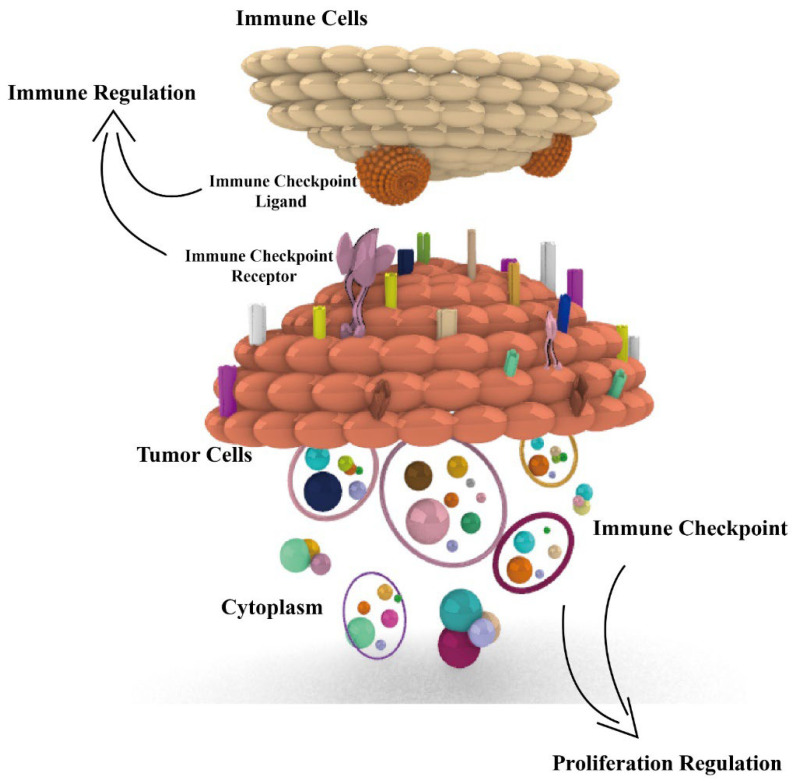
Dual immunological and proliferative regulation roles of immune checkpoints in tumors. Fifteen immune checkpoints, including PD1, PD-L1, FGL1, CD155, CD47, SIRPα, CD276, IDO1, SIGLEC-15, TIM3, Galectin-9, CD70, CD27, 4-1BBL, and HVEM, all exert dual immunological and proliferative regulation of immune checkpoints in tumors. The related proliferation regulation mechanisms are presented.

According to the report of 2022 cancer statistics [1], cancer continues to be a serious social health problem, with 1,918,030 new cases and 609,360 cancer deaths occurring in the USA. Advances in cancer treatment are essential, among which immunotherapy has become a major treatment modality in tumor therapy with immune checkpoints as the targets. However, there are still some challenges in immunotherapy, such as low response rate and few therapeutic targets. Most studies focus only on the immunomodulatory role of immune checkpoints in tumors, but we found that various immune checkpoints also play a role in proliferation regulation in tumors [2]. There are 17 pairs of immune checkpoints that we previously reviewed, with each pair consisting of a receptor and ligand (or ligands). Most of the immune checkpoints are coding protein expressed on the surface of immune cells and tumor cells, or in the cytoplasm of tumor cells. Among these, 12 pairs of immune checkpoints exert a negative immune regulatory effect, and others exert a positive regulatory effect [3]. However, the proliferative role of immune checkpoints in tumors is not fully reviewed; in this review, we aim to focus on the proliferation regulation of checkpoints and introduce immune regulation, aiming at illuminating the functions and mechanisms of immune checkpoints in tumors (Figure 1), especially for their proliferation function, with the aim of providing more therapy targets and strategies in tumor immunotherapy. 

## 2. PD1 and PD-L1

### 2.1. Biology

PD1 is a type I transmembrane protein expressed on the surface of various immune cells such as B cells, T cells, NK cells, NKT cells, and macrophages [4]. PD-L1 is ubiquitous in tumor cells such as breast cancer, gastric cancer, renal cell carcinoma (RCC), non-small cell lung cancer (NSCLC), testicular thyroid cancer, and papillary cancer [5]. The interaction between PD-1 and PD-L1 exerts a potent inhibitory immune regulation in the tumor microenvironment (TME), especially for T-cell immunity [6]. Diverse signaling pathways, which are involved in the regulation of tumor immunity, are activated upon the binding of PD1 and PD-L1. PD1 affects the metabolism of T cells by inhibiting the TCR and CD28-signaling-driven glutamine and glucose metabolism [7]. PD1 signaling increases the fatty acid oxidation and lipolysis of CD4^+^ T cells [8]. Further, the P13K-AKT-mTOR axis plays a significant role in the immune regulation of PD1/PD-L1 involved; PD1 affects the AKT pathway by inhibiting the activation of P13K (CD28 mediated) [9]. The activation of AKT increases the expression of PD-L1 and augments the inhibition of immunosurveillance [10]. Moreover, the administration of PD-L1 antibodies can effectively promote the infiltration of activated macrophages through the upregulation of the mTOR pathway [11]. Certainly, PD1/PD-L1 can affect multiple other signal pathways such as ZAP70 and RAS, as well as the inactivation of nuclear factor of activated T cells (NFAT), activator protein 1 (AP-1), and nuclear factor-κB (NF-κB), which are all involved in the activation of T cells [9].

### 2.2. Proliferative Regulatory Mechanism

**Figure 2:**
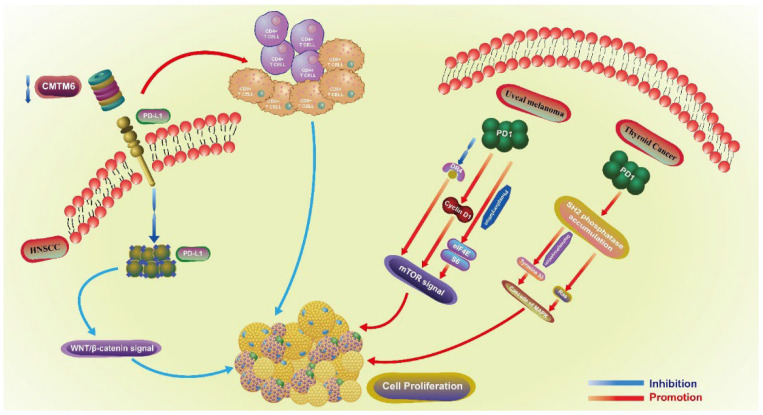
The proliferative role of PD1 in uveal melanoma and thyroid cancer, PD-L1 in HNSCC. Proliferative regulation-related pathways of PD1 involved in uveal melanoma and thyroid cancer, PD-L1 in HNSCC. Different immune checkpoints play a role in the regulation of proliferation through different signaling pathways and other molecular mechanisms in different tumors. HNSCC: head and neck squamous cell carcinoma.

Both PD1 and PD-L1 are involved in the proliferative regulation of tumors (Figure 2). In head and neck squamous cell carcinoma (HNSCC), the downregulation of CMTM6 induces a decrease in PD-L1, especially in the tumor intrinsic expression. This downregulation also further inhibits the proliferation of SCC7 cells by enhancing the infiltration of CD4^+^ and CD8^+^ T cells through the Wnt/β-catenin signaling pathway (related with the proliferation and EMT of cancer stem cells) [12], by inhibiting the translocation of β-catenin in cell nucleus [13]. In uveal melanoma, PD1 regulates the proliferation of C918 and Mum-2B melanoma cell lines. The upregulation of PD1 promotes the proliferation of C918 cell line, while the downregulation of PD1 in Mum-2B cell line exerts the opposite effect. PD1-mediated proliferation is closely related to the mTOR signaling pathway, as the overexpression of PD1 inhibits the expression of DR4 and promotes the expression of CyclinD1, as well as the phosphorylation of eIF4E and S6 [14]. In thyroid cancer, SH2 phosphatase accumulates in the cell membrane under the action of PD1, and further dephosphorylates tyrosine 32 and activates Ras, triggering the cascade of MAPK. The whole process is associated with thyroid cancer cell proliferation and migration, which is enhanced when PD1 is overexpressed, and downregulated when is blocked by siRNA and nivolumab exerts the opposite effect [15]. In NSCLC, the expression of PD-L1 is positively associated with lymph node metastasis and the expression of Ki-67, indicating that PD-L1 may boost the proliferation of NSCLC. However, this lacks sufficient mechanistic exploration [16]. Conversely, in various lung cancer cell lines, the knockdown of PD1 in H1299 and Calu-1 cells induces the phosphorylation of AKT and ERK1/2, and further promotes the proliferation of cancer cells. Overall, PD1 may serve as a tumor suppressor and inhibit the proliferation of cancer cells by activating AKT and ERK1/2 [17]. Thus, PD1 can regulate the proliferation of lung cancer cells, but whether it has a positive or negative regulatory effect needs to be further verified. Additionally, PD1^+^PD-L1^+^ T lymphoma cells promote tumor growth by activating TCR signaling [18].

Many studies have shown that various agents that block PD-1/PD-L1 are associated with proliferation inhibition. A single block of PD1 and PD-L1 can effectively inhibit proliferation and induce apoptosis of esophageal adenocarcinoma cells. This may result from a decreased expression of DNA repair genes [19]. In oral cancer cells, the treatment with *A. formosanus* extracts (AFE, 1 mg/mL) can significantly inhibit the accumulation of PD-L1 and various proliferative genes such as MMP-2, c-Myc, and Cyclin D1. This further proves that the proliferation inhibition of AFE is related to the downregulation of PD-L1 [20].

PD1 and PD-L1 are ubiquitous intrinsically within various tumors by immunohistochemical staining [15,16]. Additionally, in pancreatic cancer, it has been shown that intrinsic PD1 can effectively promote the proliferation of cancer cells; PD1 binds to MOB1 and inhibits its phosphorylation. PD1 may also affect the activation of the Hippo signaling pathway, specifically affecting CCN1 and CCN2 (the downstream of Hippo signaling pathway); the coinhibition of PD1 and Hippo exerts a more potent effect on tumor inhibition [21].

## 3. FGL1

### 3.1. Biology

LAG3 and FGL1 form a pair of immune checkpoints, with LAG3 being the most promising immune checkpoint besides PD1/PD-L1 and CTLA-4. LAG3 is a type I transmembrane protein with an extracellular region consisting of four domains—D1, D2, D3, and D4—among which D1 and D2 are responsible for binding the FD domain of FGL1, exerting an inhibitory immune regulation. LAG3 is mainly expressed on the surface of lymphocytes and stored in lysosomes, the surface of NK cells, NKT cells, CD4^+^ T cells, CD8^+^ T cells, and Treg cells [22]. The protein kinase C (PKC) signaling pathway plays a role in the translocation of LAG3 stored in the lysosomes to the surface of immune cells [23]. Furthermore, LAG3 is also expressed in the cytoplasm of various tumors such as NSCLC, breast cancer, gastric cancer, and B-cell lymphoma [24,25,26,27]. FGL1 is mainly found in tumors, including the surface of breast cancer cells and the cytoplasm of NSCLC cells [28,29]. The interaction between LAG3 and FGL1 may inhibit the proliferation of T cells and boost their exhaustion, as well as the counts and function of other immune cells including B cells, NK cells and DC cells [30]. Both LAG3 and FGL1 can affect the production of cytokines, especially TNF-α, IFN-γ, and IL-2 [28,31].

### 3.2. Proliferative Regulatory Mechanism

There are few reports about the proliferative role of LAG3 in tumors, but many studies have reported that FGL1 regulates the proliferation of various tumors either positively or negatively. FGL1 may be positively associated with the growth of gastrointestinal tumors. In MC38 colon cancer mouse models, the inhibition of FGL1 by anti-FGL1 mAb and the knockout of FGL1 in cancer cells result in a potent antitumor effect, and the proliferation of cancer cells is significantly inhibited [28]. Similarly, in SGC-7901 gastric cancer cells, the downregulation of FGL1 is correlated with a decrease in vimentin and N-cadherin and an increase in E-cadherin. This further proves that the silencing of FGL1 suppresses the proliferation of SGC-7901 gastric cancer cells [32]. Our team previously demonstrated that FGL1 is tightly associated with the proliferation of PC9 and HCC827 lung adenocarcinoma cells by regulating the cell cycle through the MYC pathway. The downregulation of FGL1 inhibits the proliferation of cancer cells, but the upstream and downstream mechanisms that are FGL1-mediated remain unclear, warranting further exploration [33]. FGL1 was also shown to be associated with cell-cycle regulation in pancreatic ductal adenocarcinoma (PDAC), as the downregulation of FGL1 promotes the arrest at the G2/M cell cycle and the expression of Cyclin B1, inhibiting the growth of tumor cells [34]. However, in the LKB1 mutant A549 lung adenocarcinoma cell line, the downregulation of FGL1 promotes the proliferation and epithelial–mesenchymal transition (EMT) of cancer cell [35]. The results are similar in hepatocellular carcinoma (HCC), as cancer cells grow faster in FGL1-null mice, which is related to the activation of Akt and mTOR signal pathways [36].

## 4. CD155

### 4.1. Biology

CD155 is a member of the nectin-like family with a similar structure to the immunoglobulin superfamily [37], which is expressed on the surface of tumor cells and immune cells such as tumor-related infiltration lymphocytes, macrophages, DC cells, melanoma cells, and pancreatic cancer cells [38,39]. There are three receptors of CD155, DNAM-1 (CD226), TIGIT, and TACTILE (CD96) [40]; the binding between CD155 and TIGIT will inhibit the cytotoxicity of NK cells and the production of IFN-γ via the PI3K signaling pathway [41,42]. CD155 is involved in T-cell metabolism; the knockdown of CD155 can promote the metabolism of T cells and increase the production of IFN-γ, and overexpression of CD155 exerts the opposite effect [43].

### 4.2. Proliferative Regulatory Mechanism

CD155 is associated with tumor growth, mainly by regulating the cell cycle and the activation of signaling pathways [40]. In medulloblastoma (MB), CD155 is a promoter of invasiveness, motility, and proliferation of MB cells; the decrease in MAP4K4 induces the downregulation of CD155 and further inhibits the growth of MB cells [44]. Similar results may be found in the three subgroups of WNTα, WNTβ, and Group 3c of MB with overexpression of c-Myc, which is positively associated with tumor proliferation [45]. In B16 melanoma, emodin can significantly inhibit the expression of CD155, and further inhibit the proliferation of tumor cells by inducing cell-cycle G2/M arrest [46]. Similarly, in B16-F10 melanoma cells, the silencing of CD155 inhibits the growth of tumor cells and simultaneously activates T cells and NK cells [47]. In hepatocellular carcinoma (HCC), CD155 also interacts with the SH2 domain of SRC (a nonreceptor tyrosine kinase) and further activates it. The CD155/SRC complex inhibits the p38 MAPK signaling pathway and further regulates the proliferation of HCC cells [48]. In cervical cancer, the suppression of CD155 activates the apoptosis and autophagy of tumor cells, inhibiting the proliferation and tumor formation by inducing G0/G1 cell-cycle arrest, through inhibiting the AKT/mTOR/NF-kB signal pathway [49]. CD155 also affects the activation of AKT in colon cancer; in CT26 and Sw620 colon cancer cells, the knockdown of CD155 inhibits the expression of Cyclin D1 and CDK4, inducing the arrest of the cell cycle in G1, further suppressing the growth of tumor cells. Furthermore, the downregulation of CD155 increases the expression of Bcl-2 and Bax, which promotes the apoptosis of colon cancer cells [50]. However, as CD155 may serve as a tumor suppressor gene in gliomas, the silencing of CD155 promotes the proliferation of SNB-19 cells, with decreased expression of Rho A/B, Rho GTPases, and Rac 1/2/3 [51].

## 5. CD47/SIRP-α

### 5.1. Biology

CD47 is a glycoprotein consisting of three domains: a short cytoplasmic region, a five-time transmembrane spanning domain, and a single extracellular V-set immunoglobulin [52]. SIRP-α is a transmembrane protein, with the extracellular region containing three immunoglobulin (Ig)-like domains, and the intracellular region containing putative tyrosine phosphorylation sites [53]. Normal cells regularly express CD47, with higher expression being found in tumor cells such as breast cancer, hepatocellular carcinoma, ovarian clear cell carcinoma, and melanoma [54]. SIRP-α expression was found on the surface of a variety of immune cells, including monocytes, macrophages, and myeloid DCs [55]. CD47 can bind to the extracellular IgV domain of SIRP-α and can induce the tyrosine phosphorylation on the intracellular ITIM motif. Both CD47 and SIRPα can inhibit macrophage-mediated phagocytosis [54,55].

### 5.2. Proliferative Regulatory Mechanism

Both CD47 and SIRP-α regulate apoptosis and proliferation in various tumors. In prostate cancer, the overexpression of SIRP-α induces the apoptosis of prostate cancer cells, and its silencing exerts the opposite effect. However, SIRP-α inhibits the activation of the NF-κB pathway and p38 mitogen-activated protein kinase, and further suppresses the expression of cyclooxygenase-2 (COX-2) [56]. In nasopharyngeal carcinoma (NPC), the coinhibition of miR-200a and CD47 exerts a more potent inhibition of tumor proliferation than the single-block group, but the specific mechanism has not been elucidated clearly [57]. In adamantinomatous craniopharyngioma (APC), CD47 activates the MAPK/ERK pathway and thus promotes the proliferation, invasion, and migration of APC cells. The silencing of CD47 induces microglia-mediated phagocytosis and the inhibition of growth of tumor cells [58]. In colorectal cancer (CRC), CD47 is positively associated with the proliferation and metastasis of CRC cells. CD47 exerts the proliferation regulation intrinsically of CRC cells to bind with ENO1, protecting it from ubiquitin-mediated degradation, and inducing the phosphorylation of ERK and glycolytic activity of CRC cells [59]. The activation of CD47 promotes the proliferation of U87 and U373 astrocytoma cells, and CD47 may interact with PLIC-1, affecting the activation of PI3K/Akt pathway to stimulate the growth of astrocytoma cells [60]. Many studies indicate that anti-CD47 therapy can significantly enhance the treatment effect of chemotherapy and inhibit the growth of tumors; in HCC, the administration of B6H12 (anti-CD47 mAb, 10 µg/mL) was shown to augment the effect of doxorubicin and cisplatin in HCC treatment, inhibiting the macrophage-mediated phagocytosis. The combination of anti-CD47 antibody (400 µg/mouse) with doxorubicin (2 mg/kg) in a mouse model was shown to significantly inhibit tumor growth [61]. Similar results can be found in breast cancer, as the treatment with of an anti-CD47 antibody promotes the effect of doxorubicin chemotherapy, inhibiting the growth of tumor cells significantly [62].

## 6. CD276

### 6.1. Biology

CD276 is a type I transmembrane protein with two extracellular IgC and variable (IgV) domains [63], mainly expressed on tumor cells, including breast cancer, renal cell carcinoma, NSCLC, endometrial cancer, and ovarian cancer cells [64]. CD276 is an immune checkpoint that is synergized with other immune checkpoints such as PD1 and CTLA-4, and in both innate and adaptive immunity can be modulated by CD276 [65]. CD276 on the cell surface can protect cells from lysis by NK cells and increase the production of cytokines from macrophages by Toll-like receptor 4 and 2 [66,67]. CD276 can also inhibit the proliferation and activation of CD4+ and CD8+ T cells by inhibiting the production of cytokines such as IL-2 and IFN-γ, which are associated with the inactivation of NK-κB, NFAT, and AP-1 [68].

### 6.2. Proliferative Regulatory Mechanism

CD276 mainly regulates the proliferation of oral cancer, hepatocellular carcinoma, colorectal cancer, hematologic tumor, gynecological oncology, and lung adenocarcinoma. In oral squamous cell carcinoma (OSCC), CD276 is involved in tumor proliferation regulation, mainly via glycometabolism. The knockdown of CD276 inhibits tumor growth, as it was also found that CD276 in Ca9-22 oral cancer cells can better interact with DC-SIGN with terminal α-galactose and higher fucosylation. The structure differences in CD276 in OSCC may induce the proliferation regulation in OSCC [69]; in Cal27 and SCC25 OSCC cell lines, CD276 upregulates the expression of HIF-1α via the PI3K/Akt/mTOR pathway, increases the uptake of glucose, and then enhances the Warburg effect, further promoting tumor growth [70]. In HCC, CD276 affects the activation of the PI3K/AKT/MMPs pathway, as the downregulation of CD276 inhibits the expression of MMP2, MMP14, and the activation of MMP2, as well as the phosphorylation of AKT, which promotes HCC growth, invasion, and migration, involved in vasculogenic mimicry formation [71]. In CRC, miRNA and NF-κB pathway jointly mediate the regulation of tumor proliferation. It has been shown that miRNA-128 inhibits the expression of CD276, and high levels of CD276 promote tumor growth [72]; furthermore, CD276 promotes tumor angiogenesis and increases the expression of VEGFA by activating the NF-κB pathway [73]. In hematologic tumors, especially in acute monocytic leukemia and mantle cell lymphoma (MCL), CD276 induces cell-cycle arrest in the G0/G1 phase, and xenograft models show the potent inhibitory role of CD276 in tumor growth. In the U937 acute monocytic leukemia cell line, the knockdown of CD276 is related to the decrease in PCNA and Ki67, inducing cell-cycle arrest in the G0/G1 phase, with a mean tumor growth rate of just 59.4% in xenograft models compared to the control group [74]. Similarly, in MCL Z138 and Maver mantle cell lymphoma cell lines, the silencing of CD276 induces an arrest in the G0/G1 phase, inhibiting tumor proliferation. This was confirmed by CCK8 assay and xenograft models and by comparing the results to control groups; the tumor growth rate was just 59.1% in the Maver cell model and 65.0% in the Z138 cell model [75]. The AKT signaling pathway is the key regulator in ovarian and cervical cancer proliferation. In cervical cancer, miRNA-199a targets the 3′-untranslated region of CD276 and regulates its expression, and then activates the AKT/mTOR signaling pathway to inhibit tumor proliferation [76]. Additionally, CD276 also inhibits tumor growth in ovarian cancer via activating the PI3K/AKT signaling pathway through the upregulation of BCL-2, which is related to chemoresistance [77]. In LUAD, Ets-like protein 1 (ELK1) works as a transcription factor that binds to CD276 and regulates its expression, further promoting tumor proliferation and EMT process in the A549 cell line. However, the specific mechanisms behind this effect warrant further exploration [78,79].

## 7. IDO-1

### 7.1. Biology

Inherently, IDO1 is a type of rate-limiting enzyme, and works in converting tryptophan to kynurenines. IDO1 is always expressed on the surface of antigen-presenting cells, myeloid-derived suppressor cells (MDSCs), and various tumor cells, including sarcoma, breast cancer, and chronic lymphocytic leukemia [80]. The activation of GCN2 and mTOR signals are associated with the inactivation of T cells, as higher expression of Kyn boosts the necrosis of T cells and the conversion of CD4+T cells into Tregs [80]. Further, multiple signals related to inhibitory immune regulation involve IDO1, including JAK/STAT, NF-κB, TGF-β, and PKC [81,82].

### 7.2. Proliferative Regulatory Mechanism

IDO1 promotes the progression of various tumors via diverse signaling pathways. In CRC, the IDO1–kynurenine pathway (KP) is directly associated with the proliferation of tumors, as the metabolites from the pathway induce the activation of the PI3K-Akt signal to enhance the nuclear translocation of β-catenin, which is correlated with CRC proliferation and apoptosis resistance [82]. In HCC, the suppression of IDO1 inhibits the proliferation and motility of HCC cells. IDO1 also induces the activation of the Aryl hydrocarbon receptor (AhR), which further activates the Src-PTEN-PI3K/Akt-GSK-3β signal, which is associated with the activity of nuclear translocation of β-catenin, which is finally involved in the proliferation of HCC cells [83]. Similarly, in colon cancer, the silencing of IDO1 inhibits the expression of β-catenin in the nucleus, as well as the expression of Axin2 and Cyclin D1, further exerting an inhibitory effect on tumor proliferation [84].

## 8. SIGLEC-15

### 8.1. Biology

SIGLEC-15 is a member of the Siglec gene family. SIGLEC-15 binds to Sialyl-Tn antigen with the help of the sialic acid-binding immunoglobulin-type lectin structure [85]. SIGLEC-15 is shown to be highly expressed on the surface of myeloid cells and in several tumor cells such as bladder cancer, kidney cancer, endometrioid cancer, osteosarcoma, lung cancer, and thyroid cancer [86,87]. There are two SIGLEC-15 fusion proteins—hS15-hIg and mS15-mIg—and both inactivate T cells. Additionally, SIGLEC-15 is synergistic with PD1 and PD-L1, and is associated with inhibition of the antigen-specific T-cell response and the secretion of IFN-γ [85].

### 8.2. Proliferative Regulatory Mechanism

SIGLEC-15 may be involved in tumor proliferation regulation, but more evidence is warranted for further validation. In MNNG/HOS and 143B osteosarcoma cells, the overexpression of SIGLEC-15 promotes the proliferation, migration, and invasion of tumor cells. The silencing of SIGLEC-15 exerts the opposite effect, as the expression of dual-specificity phosphatase 1 (DUSP1) is downregulated conformably, and the JNK/MAPK and p38/MAPK signals are both activated simultaneously [88]. Similarly, in osteosarcoma, another signal of STAT3/Bcl-2 is also shown be involved in tumor proliferation, and the silencing of SIGLEC-15 induces the upregulation of apoptosis- and pyroptosis-related proteins. This shows that SIGLEC-15 inhibits tumor proliferation by affecting the activity of the STAT3/Bcl-2 signaling pathway, thereby affecting the apoptosis and pyroptosis of osteosarcoma cells [89]. Through CCK8 and colony formation assay, SIGLEC-15 demonstrates involvement in NSCLC cell proliferation. The knockdown of SIGLEC-15 inhibits tumor proliferation, but the underlying mechanisms remain unclear currently [90].

## 9. TIM3 and Galectin-9

### 9.1. Biology

TIM3 is a member of the TIM family and is characterized by five tyrosine residues in the cytoplasmic domain [91]. Galectin-9 is a kind of tandem-repeat-type galectin and is characterized by two carbohydrate recognition domains, and was first recognized as a factor associated with eosinophil chemoattractant and activation [92,93]. High expression of TIM3 is found on the surface of immune cells such as monocytes, NK, DC, CD4+, and CD8+ T cells [91]. Galectin 9 is widely distributed in many tissues such as the lung, liver, skeletal muscle, and cardiac muscle [94]. The interaction between TIM3 and Galectin-9 creates an inhibitory tumor microenvironment. Galectin-9 can induce apoptosis and inactivation of T cells, as its ligand TIM3 significantly inhibits the response of T cells and boosts the exhaustion of CD8+ T cells, promoting the expansion of MDSCs and Tregs [94,95].

### 9.2. Proliferative Regulatory Mechanism

Both TIM3 and Galectin-9 are potent proliferative regulatory factors in various tumors, simultaneously affecting the function of migration and invasion while regulating the content of matrix metalloproteinase (MMP), N-cadherin, and E-cadeherin. In esophageal squamous cell carcinoma (ESCC), it was found that the downregulation of TIM3 inhibits the invasion, migration, and proliferation of tumors. Diverse mechanisms are involved in this regulation, as the expression of TIM3 decreases and the content of MMP-9 and E-cadherin increases, while that of TIMP-1, vimentin, and N-cadherin decreases with the inactivation of p-Akt, p-GSK-3β, and SNAIL pathways [96]. Similarly, in nasopharyngeal carcinoma, the overexpression of TIM3 promotes tumor proliferation, migration, and invasion, with higher levels of MMP9, MMP2, vimentin, and N-cadherin, lowering the expression of E-cadherin. Moreover, the expression of SMAD7 is inhibited, but SMAD2 and SNAIL1 are increased, indicating that TIM3 exerts the proliferation, migration, and invasion regulation mainly through the SMAD7/SMAD2/SNAIL1 axis [97].

NF-κB signaling pathway is a critical regulator in TIM3 proliferation regulation. The overexpression of TIM3 in breast cancer induces the upregulation of various factors, including VEGF, TWIST, MMP1, c-Myc, and Cyclin D1, which promote tumor proliferation, tubal formation, invasion, migration, and tight junction deterioration. Further, the NF-κB/STAT3 signaling pathway is activated and E-cadeherin is downregulated during the regulation [98]. In osteosarcoma, the silencing of TIM3 also inhibits the activation of NF-κB signals. Moreover, as the expression of vimentin and SNAIL decreases, the level of NF-κB p65 phosphorylation increases, and the proliferation of MG-63 osteosarcoma cells is significantly inhibited. This indicates that the downregulation of TIM3 mainly affects the activation of the NF-κB/SNAIL signaling pathway [99]. In myeloma U266 and RPMI-8226 cell lines, it was concluded that the high expression of TIM3 is associated with tumor proliferation and apoptosis. The knockdown of TIM3 alters a variety of pathways, including AKT, P13K, mTOR, and NF-κB, which are all downregulated, resulting in the inhibition of tumor proliferation and increased apoptosis [100].

Galectin-9 exerts a proliferation regulation role in various gastrointestinal tumors. Galectin-9 plays an important role in the regulation of cell apoptosis. In esophageal adenocarcinoma (EAC), Galectin-9 regulates the proliferation of SK-GT4, OE33, OE19, and OACM5.1c cell lines via inducing cell apoptosis and autophagy, and by affecting the cell cycle. The administration of Galectin-9 elevates the level of cleaved PARP and cleaved caspase-3/9, consequently inducing the apoptosis of EAC cells. Furthermore, the autophagy markers SQSTM1/p62 and LC3-II are both upregulated, and cell-cycle-related molecules such as CDK4, Cyclin D1, and Cyclin E are all decreased during treatment with Galectin-9 [101]. Similar results are obtained in ESCC; the mouse model also displays the proliferative role of Galectin-9 in ESCC, as its administration was also shown to inhibit tumor growth by inducing cell apoptosis via activating caspase-3, p38 mitogen-activated protein kinase, and JNK [102]. 

The upregulation of expression of caspase-cleaved keratin 18 (CCK18, a marker of cell apoptosis) is one of the characteristics of Galectin-9 in regulating the proliferation of various gastrointestinal tumors. In cholangiocarcinoma, Galectin-9 induces cell apoptosis and inhibits the proliferation of TFK-1 and HuH-28 cholangiocarcinoma cell lines, with an increase in CCK-18 and cytochrome c [103]. In colon cancer, the treatment with Galectin-9 also upregulates the expression of CCK-18 and induces the apoptosis of CACO-2 and CW-2 cell lines. It also increases the expression of IL-18 and tissue inhibitor of metalloproteinases 2 (TIMP-2) in WiDr cells. In addition, Galectin-9 induces CW-2 cells to move into G0/G1 cycle arrest, and exerts in tumor growth inhibition [104]. In liver metastatic carcinoma from pancreatic cancer cell lines KMP2, KMP7, and KMP8, the expression of CCK18, fluorescein isothiocyanate (FITC), cleaved caspase-3, cleaved PARP, caspase-7, Smac/Diablo, HtrA2/Omi, and cytochrome c is increased. Forty-two miRNAs are differentially expressed during the administration of Galectin-9. However, no effect on cell-cycle-related proteins is established, indicating that Galectin-9 induces cell apoptosis, but does not affect the cell cycle in tumor proliferation [105]. Similar results can be identified in gallbladder carcinoma (GBC), as CCK18 and phosphorylated p53 are upregulated during the administration of Galectin-9. Sixty-six miRNAs are differently expressed; similarly, there is no effect on cell-cycle-related proteins [106].

### 9.3. Proliferation Regulation of Other Immune Checkpoints

CD70/CD27 are mainly involved in lymphoma/leukemia proliferation regulation. Particularly, blocking CD70 promotes the proliferation of leukemic cells during the remission phase [107]. In acute myeloid leukemia (AML), blocking the interaction between CD70/CD27 may promote the asymmetric differentiation and division of AML cells, further inhibiting tumor proliferation [108]. The knockout of 4-1BBL, the ligand of 4-1BB, promotes the accumulation of Gsk3β in the nucleus of colon cancer cells and inhibits the expression of Wnt signal pathway-related genes, inhibiting the proliferation of colon cancer cells [109]. In ovarian cancer, the overexpression of HVEM (TNFRSF14), a member of the TNF receptor superfamily, is associated with the activation of AKT/mTOR signaling, promoting the expression of Bcl-2 and HIF-1α, which is positively associated with tumor proliferation and negatively related to cell apoptosis [110].

### 9.4. Perspective

Immune checkpoints have become the major targets in tumor immunotherapy. PD1 and PD-L1 are mature treatment targets, but the response rate is just about 30% in lung cancer immunotherapy [111]. Furthermore, the immune-related adverse events rate in anti-CTLA-4 treatment is high, reaching up to 70% in various tumors [112]. Thus, we can consider that the specific mechanism for each immune checkpoint in tumors has not been elucidated clearly; the role of their receptors or ligands in tumor proliferation regulation is still unknown, and we consider the enormous potential in this area. In this review, we present the proliferative role of 15 immune checkpoints, including PD1, PD-L1, FGL1, CD155, CD47, SIRPα, CD276, IDO1, SIGLEC-15, TIM3, Galectin-9, CD70, CD27, 4-1BBL, and HVEM, in tumor progression. The related mechanisms have been folded into Table 1. Our findings aim to provide more therapy targets and elicit more research directions in tumor treatment.

Immune checkpoints may not only serve as an immune regulators but as proliferation regulators, especially for intrinsic tumor genes [48,59], which are more likely to play an indispensable role in the regulation of tumor proliferation [21,113]. Most immune checkpoints, especially Cyclin D1 [14,50,84], are positively associated with he regulation of tumor proliferation, influencing the cell cycle. In addition, cell apoptosis is increased simultaneously [19,50,56], affecting the activity of diverse signal pathways, including P13K [100], AKT [10], mTOR [9], and NK-κB [56]. 

Furthermore, PD1/PD-L1 serve as a pair of mature immune checkpoints in tumor immunotherapy. In fact, their proliferative role has been proven to be present in various tumors, Similarly, FGL1 and CD155 can regulate the cell cycle and exert their function by activating the AKT, mTOR, and MYC signal pathways. CD47/SIRPα may affect tumor growth intrinsically. Anti-CD47 therapy was shown to promote the effect of chemotherapy by inhibiting tumor proliferation. The number of studies on CD276 is the largest among the 15 immune checkpoints. On the other hand, the number of studies regarding IDO1 and SIGLEC-15 is the lowest, indicating that the research potential of these two immune checkpoints is significant. NK-κB is the critical signal pathway in TIM3 regulation. Galectin-9 mainly plays a role in growth regulation in gastrointestinal tumors, characteristically by upregulating CCK-18. CD70/CD27 has been found to be involved in hematologic tumors, 4-1BBL in colon cancers, and HVEM in ovarian cancers. However, whether these genes play a role in other tumors is still unknown, warranting further exploration.

Interestingly, only PD1/PD-L1, CD47/SIRPα, TIM3/Galectin-9, and CD70/CD27 checkpoint have proved to work in pairs. All other checkpoints work individually. Whether other pairs of checkpoints work in tumor proliferation is unknown and warrants further exploration.

Our review elucidates the proliferative role of immune checkpoint genes and their immune regulation, elaborating the diverse mechanisms of immune checkpoints on carcinogenesis in various tumors, providing novel research insight and possible theoretical basis for the occurrence and development of tumors. Most importantly, PD1/PD-L1 serves as a milestone in tumor immunotherapy, and also regulates proliferation in tumors, if there are immune checkpoints that exert a similar function of regulation of tumor proliferation, this particular function may act as a link that provides a strong theoretical basis to develop clinical combination therapy in the future. In this post-PD1 era, more therapy targets may be discovered in tumor therapy with the proliferative regulation by immune checkpoints opening a whole novel epoch for tumor combination therapy.

## 10. Conclusions

Various immune checkpoints are emerging in this post-PD1 era, with a huge potential to be immunotherapy targets. However, the specific mechanisms of these immune checkpoints are not fully elucidated. Therefore, we have reviewed the literature on the mechanism of seventeen pairs of immune checkpoints and found that fifteen immune checkpoints (PD1, PD-L1, FGL1, CD155, CD47, SIRPα, CD276, IDO1, SIGLEC-15, TIM3, Galectin-9, CD70, CD27, 4-1BBL, and HVEM) exert proliferative and immunologic regulatory effects in tumors. This review mainly focuses on the regulation of proliferation that immune checkpoints mediate, and it is found that immune checkpoints mostly affect cell cycle and apoptosis by affecting some classical signaling pathways, and finally regulate cell proliferation. The most significant finding of this review is that on the one hand, the dual regulation function of the immune checkpoints is able to provide a new direction and insight for research on immune checkpoints, and on the other hand, owing to the role of PD1/PD-L1 that also functions to regulate tumor proliferation, it can provide the theoretical basis for developing clinical combination therapy in the future.

## Figures and Tables

**Table 1:** Molecular mechanisms of immune checkpoints regulating tumor proliferation.

Immune Checkpoint	Tumor	Molecular Mechanisms	PMID
PD1/PD-L1	Head and neck squamous cell carcinoma	①The downregulation of CMTM6 inhibits the expression of PD-L1 ②The decrease in PD-L1 further inhibits SCC7 cell proliferation by Wnt/β-catenin signaling pathway ③The decrease in PD-L1 induces the infiltration of CD4+ and CD8+ T cells	24871033
	Uveal melanoma	①PD1 promotes the expression of CyclinD1 and mTOR signaling pathway ②PD1 inhibits the expression of DR4	32686562
	Thyroid cancer	①PD1 promotes the accumulation SH2 phosphatas ②The accumulation SH2 phosphatase dephosphorylates tyrosine 32 and activates Ras, triggering the cascade of MAPK	33413561
	Non-small cell lung cancer	Expression of PD-L1 is positively associated with lymph node metastasis and the expression of Ki-67	30769852
	Non-small cell lung cancer	The downregulation of PD1 induces the phosphorylation of AKT and ERK1/2	32161124
	T lymphoma	PD1+PD-L1+ T lymphoma cells promote tumor growth by activating TCR signaling	34157461
	Esophageal adenocarcinoma	A single block of PD1 and PD-L1 decrease the expression of DNA repair genes	35228614
	Oral cancer	The proliferation inhibition of AFE is related to the downregulation of PD-L1	30116189
FGL1	MC38 colon cancer mouse models	The knockout of FGL1 inhibits the proliferation of cancer cells	30580966
	SGC-7901 gastric cancer cells	The downregulation of FGL1 is correlated with a decrease in vimentin and N-cadherin and an increase in E-cadherin	29845203
	Lung adenocarcinoma	The downregulation of FGL1 inhibits the proliferation of PC9 and HCC827 cancer cells via MYC pathway	35399566
	LKB1 mutant A549 lung adenocarcinoma cell line	The downregulation of FGL1 promotes the proliferation and epithelial–mesenchymal transition (EMT) of cancer cell	31322182
	Pancreatic ductal adenocarcinoma	The downregulation of FGL1 promotes the arrest at the G2/M cell cycle and the expression of Cyclin B1, inhibiting the growth of tumor cells	33801246
	Hepatocellular carcinoma	Cancer cells grow faster in FGL1-null mice with the activation of Akt and mTOR signal pathways	26225745
CD155	Medulloblastoma	The decrease in MAP4K4 induces the downregulation of CD155 and further inhibits the growth of Medulloblastoma cells	35296518
	B16 melanoma	Emodin inhibits the expression of CD155 and further inhibits the proliferation of tumor cells by inducing cell-cycle G2/M arrest	31325728
	B16-F10 melanoma	The silencing of CD155 inhibits the growth of tumor cells and activates T cells and NK cells	33089844
	Hepatocellular carcinoma	The CD155/SRC complex inhibits p38 MAPK signaling pathway and further regulates the proliferation of HCC cells	35384345
	Cervical cancer	The suppression of CD155 ①activates the apoptosis and autophagy of tumor cells, ②induces G0/G1 cell-cycle arrest ③inhibits the AKT/mTOR/NF-kB signal pathway	34164340
	CT26 and Sw620 colon cancer	The suppression of CD155 ①inhibits the expression of Cyclin D1 and CDK4, ②induces the arrest of the cell cycle in G1	28816021
	Glioma	The silencing of CD155 decreases the expression of Rho A/B, Rho GTPases, and Rac 1/2/3	22363471
CD47/SIRP-α	Prostate cancer	The overexpression of SIRP-α ①induces the apoptosis of prostate cancer cell ②inhibits the activation of NF-κB pathway and p38 mitogen-activated protein kinase ③suppresses the expression of cyclooxygenase-2 (COX-2)	28588738
	Nasopharyngeal carcinoma	The coinhibition of miR-200a and CD47 exerts a more potent inhibition of tumor proliferation than the single-block group	32149101
	Adamantinomatous craniopharyngioma (APC)	CD47 activates the MAPK/ERK pathway	35156226
	Colorectal cancer (CRC)	①CD47 binds with ENO1 and protects it from ubiquitin-mediated degradation ②CD47 induces the phosphorylation of ERK and glycolytic activity of CRC cells	32226539
	U87 and U373 astrocytoma	CD47 interacst with PLIC-1 and e activates PI3K/Akt pathway	21125662
	Hepatocellular carcinoma	B6H12 (anti-CD47 mAb) augments the effect of doxorubicin and cisplatin in HCC treatment, inhibiting the macrophage-mediated phagocytosis.	26351778
	Breast cancer	Anti-CD47 antibody promotes the effect of doxorubicin chemotherapy, inhibiting the growth of tumor cells significantly	30056566
CD276	Oral squamous cell carcinoma (OSCC)	①CD276 interacts with DC-SIGN with terminal α-galactose and higher fucosylation ②The knockdown of CD276 inhibits tumor growth	26438868
	Oral squamous cell carcinoma (OSCC)	①CD276 upregulates the expression of HIF-1α via the PI3K/Akt/mTOR pathway, ②CD276 increases the uptake of glucose and enhances the Warburg effect	31737114
	Hepatocellular carcinoma	The downregulation of CD276 inhibits the expression of MMP2, MMP14, and the activation of MMP2	33204103
	Colorectal cancer	miRNA-128 inhibits the expression of CD276, and high levels of CD276 promote tumor growth	33743142
	Colorectal cancer	CD276 promotes tumor angiogenesis and increases the expression of VEGFA by activating the NF-κB pathway	31974361
	Acute monocytic leukemia and mantle cell lymphoma	The knockdown of CD276 is related to the decrease in PCNA and Ki67, inducing cell-cycle arrest in G0/G1 phase	26203263
	MCL Z138 and Maver mantle cell lymphoma	The silencing of CD276 induces an arrest of the G0/G1 phase	25872657
	Cervical cancer	miRNA-199a targets the 3′-untranslated region of CD276 and regulates its expression, and then activates the AKT/mTOR signaling pathway to inhibit tumor proliferation	32856542
	Ovarian cancer	CD276 activates the PI3K/AKT signaling pathway through the upregulation of BCL-2	31819652
	Lung adenocarcinoma	ELK1 works as a transcription factor that binds to CD276 and regulates its expression, further promoting tumor proliferation and EMT process in the A549 cell line	30127617 34970415
IDO1	Colorectal cancer	Metabolites from the IDO1–kynurenine pathway induce the activation of the PI3K-Akt signal to enhance the nuclear translocation of β-catenin and promotes CRC proliferation and apoptosis resistance	30679179
	Hepatocellular carcinoma	①IDO1 induces the activation of Aryl hydrocarbon receptor ②IDO1 activates the Src-PTEN-PI3K/Akt-GSK-3β signal	34769098
	Colon cancer	The silencing of IDO1 ①inhibits the expression of β-catenin in the nuclears ②inhibits the expression of Axin2 and Cyclin D1	23669411
SIGLEC-15	MNNG/HOS and 143B osteosarcoma cells	The silencing of SIGLEC-15 induces the decreased expression of dual-specificity phosphatase 1 and the activation of JNK/MAPK and p38/MAPK signals	34336699
	Osteosarcoma	SIGLEC-15 inhibits tumor proliferation by affecting the activity of STAT3/Bcl-2 signaling pathway	35398779
TIM3	Esophageal squamous cell carcinoma	The downregulation of TIM3 ①increases the expression of MMP-9 and E-cadherin ②TIMP-1, vimentin, and N-cadherin decreases ③inhibits the activation of p-Akt, p-GSK-3β, and SNAIL pathways	27430162
	Nasopharyngeal carcinoma	TIM3 exerts proliferation, migration, and invasion regulation, mainly through the SMAD7/SMAD2/SNAIL1 axis	32184631
	Breast cancer	The overexpression of TIM3 ①induces the upregulation of VEGF, TWIST, MMP1, c-Myc, and Cyclin D1 ②promotes the activation of NF-κB/STAT3 signaling pathway ③induces the downregulation of E-cadeherin	33223752
	Osteosarcoma	The silencing of TIM3 ①inhibits the activation of NF-κB signals ②decreases the expression of vimentin and SNAIL ③increases the level of NF-κB p65 phosphorylation	27706678
	U266 and RPMI-8226 myeloma cell lines	The knockdown of TIM3 induces the downregulation of AKT, P13K, mTOR, and NF-κB, resulting in the inhibition of tumor proliferation and increased apoptosis	33330064
Galectin-9	Esophageal adenocarcinoma	The administration of Galectin-9 ①elevates the level of cleaved PARP and cleaved caspase-3/9 ②autophagy markers SQSTM1/p62 and LC3-II are both upregulated ③CDK4, Cyclin D1, and Cyclin E are all decreased	28586026
	Esophageal squamous cell carcinoma	The administration of Galectin-9 inhibits tumor growth by inducing cell apoptosis via activating caspase-3, p38 mitogen-activated protein kinase, and JNK	31146370
	Cholangiocarcinoma	Galectin-9 induces cell apoptosis and inhibits the proliferation of TFK-1 and HuH-28 cholangiocarcinoma cell lines with an increase in CCK-18	26260906
	Colon cancer	①Galectin-9 upregulates the expression of CCK-18 ②Galectin-9 induces the apoptosis of CACO-2 and CW-2 cells ③Galectin-9 increases the expression of IL-18 and TIMP-2	33907832
	Liver metastatic carcinoma from pancreatic cancer cell lines KMP2, KMP7, and KMP8	The administration of Galectin-9 induces the increase in CCK18, fluorescein isothiocyanate (FITC), cleaved caspase-3, cleaved PARP, caspase-7, Smac/Diablo, HtrA2/Omi, and cytochrome c	28656219
	Gallbladder carcinoma	The administration of Galectin-9 induces the increase in CCK18 and phosphorylated p53	26797414

## Abbreviations

PD1/PD-L1 (Programmed Cell Death 1/Programmed Cell Death Ligand 1); RCC (renal cell carcinoma); NSCLC (non-small cell lung cancer); TME (tumor microenvironment); NFAT (nuclear factor of activated T cells); AP-1 (activator protein 1); NF-κB (nuclear factor-κB); HNSCC (head and neck squamous cell carcinoma); AFE (*A. formosanus* extracts); PKC (protein kinase C); PDAC (pancreatic ductal adenocarcinoma); EMT (epithelial–mesenchymal transition); HCC (hepatocellular carcinoma); MB (medulloblastoma); Ig (immunoglobulin); COX-2 (cyclooxygenase-2); NPC (nasopharyngeal carcinoma); APC (adamantinomatous craniopharyngioma); CRC (colorectal cancer); OSCC (oral squamous cell carcinoma); MCL (mantle cell lymphoma); ELK1 (Ets-like protein 1); MDSCs (myeloid-derived suppressor cells); KP (kynurenine pathway); AhR (Aryl hydrocarbon receptor); DUSP1 (dual-specificity phosphatase 1); MMP (matrix metalloproteinase); ESCC (esophageal squamous cell carcinoma); EAC (esophageal adenocarcinoma); CCK18 (caspase-cleaved keratin 18); TIMP-2 (tissue inhibitor of metalloproteinases 2); FITC (fluorescein isothiocyanate); GBC (gallbladder carcinoma); AML (acute myeloid leukemia).
